# Pediatric pleuropulmonary blastoma: analysis of four cases

**DOI:** 10.1186/s12885-024-12977-1

**Published:** 2024-10-11

**Authors:** Hana Hemead, Rania Gaber Aly, Mostafa Kotb, Ahmed Abdelaziz

**Affiliations:** 1 Lecturer of Cardiothoracic Surgery, Alexandria Faculty of Medicine, Alexandria, Egypt; 2 Lecturer of Pathology, Alexandria Faculty of Medicine, Alexandria, Egypt; 3 Lecturer of Pediatric Surgery, Alexandria Faculty of Medicine, Alexandria, 21615 Egypt

**Keywords:** Lung cancer, Pediatrics, Pleuropulmonary blastoma

## Abstract

**Background:**

Pleuropulmonary Blastoma (PPB) is an extremely uncommon, highly aggressive tumor that arises from either the lungs or pleura. According to Dehner, PPB was classified into three groups: type I (cystic), type II (mixed), and type III (solid). Type I tends to occur more commonly in infants and has a more favorable prognosis compared to types II and III. This tumor is very rare in pediatric age group; hence, there is no consensus on the optimal treatment regimen for it to date. Type I tumors, which resemble congenital lung cysts, can eventually progress to more aggressive type II and type III tumors. This article aims to increase general awareness of this pathology, clinical presentation, and differential diagnosis in order to identify this rare entity early in its course. By presenting 4 such cases, we highlight that PPB can be missed early in diagnosis and it is important to be alert when putting this rare tumor in differential diagnosis of cystic lung lesions.

**Methods:**

A retrospective study was conducted between 2015 and 2020 involving patients who had a definitive diagnosis of PPB with emphasis on clinical presentation, preoperative imaging studies, intra-operative findings, pathological reports, ancillary treatment, and outcomes. All patients were followed up every 6 months to monitor local recurrence and distant metastasis by undergoing physical exam and non-contrast enhanced CT of the chest. The primary outcome is to identify the mortality and morbidity (recurrence and distant metastasis) of PPB for cases admitted in our institute.

**Results:**

Four children were diagnosed with PPB during the study period. Clinically, patients presented with manifestations ranging from respiratory distress, fever to obstructive shock and radiologically, 2 cases were presented with mediastinal mass and the other 2 presented with pneumothorax. Regrettably, none of the cases were diagnosed pre-operatively. One lesion proved to be type I, 2 were type II and one was type III. All cases underwent chemotherapy using the combination of vincristine, Adriamycin and cyclophosphamide (VAC regimen). Recurrence was detected in a type II case, around 2 years after operation, and the other type II case developed brain metastasis that was discovered 3 years after operation. Type I case showed no local or distant metastasis.

**Conclusion:**

A prompt preoperative diagnosis and workup of cases of PPB is crucial to enable optimal intervention intraoperatively and early postoperative treatment. Though it is uncommon, PPB should be considered in the differential diagnosis of cystic lung lesions.

## Introduction

Pleuropulmonary Blastoma (PPB) is very rare, highly aggressive tumor that originates from either the lungs or pleura [1]. In 1988, Manivel et al. first reported this rare condition in a study involving 11 patients [2]. Children aged below six years are more commonly affected [1, 3]. In 1995, Dehner further classified PPB into three groups: type I (cystic), type II (mixed), and type III (solid) [3]. Compared to the last 2 subtypes, which have median diagnostic ages of 34 and 44 months, respectively, the former type typically affects infants (median diagnosis age of 10 months) [4]. The prognosis for type I is generally better than that of types II and III [5]. Morphologically, type II resembles Wilms tumor; thus, sometimes incorrectly called “extra-renal Wilms’ tumor” [6]. Type I PPB children are treated with surgical resection either with or without adjuvant chemotherapy [7]. There is no consensus on the optimal treatment regimen for PPB to date since this entity is extremely uncommon in pediatric age group.

Type I tumors, which resemble congenital lung cysts, have the potential to progress into the more aggressive type II and type III tumors. For this reason, this article aims to increase general awareness of this pathology, clinical presentation, and differential diagnosis in order to identify this rare pathology as early as possible in its course. Hence, prompt histological diagnosis and differentiation from other benign cysts as well as congenital airway malformations is crucial. Additionally, this tumor must be included in the differential diagnosis of a child with unexplained spontaneous pneumothorax and in the radiological differential of a child with total opacification of the entire hemithorax. By presenting such 4 cases, we highlight that PPB can be missed early in diagnosis and it is important to be alert when putting this rare tumor in differential diagnosis.

## Methods

After approval of the ethical committee of our institute, a retrospective study was conducted between 2015 and 2020. Data was collected from patients who had a definitive diagnosis of PPB. This includes history taking and clinical presentation at time of admission. Emphasis was given to the preoperative symptoms such as dyspnea, respiratory distress, hemoptysis and chest pain. Clinical examination focused on detecting signs of respiratory distress and manifestations of metastasis such as weight loss and even neurologic complains. Preoperative chest X-rays and CT chest were retrieved and analyzed for chest masses or pneumothorax. Details of intra-operative findings and maneuvers done amid surgical intervention were recorded. Data retrieval included pathological reports using hematoxylin and eosin (H & E) as well as immunohistochemistry (IHC). Adjuvant chemotherapy and outcomes in the form of mortality, recurrence and distant metastasis were recorded as well. We excluded cases with indefinite diagnosis of PPB and those who lost during follow-up period.

All patients were followed up every 6 months to monitor local recurrence and distant metastasis by undergoing physical exam and non-contrast enhanced CT of the chest. The primary outcome is to identify the mortality and morbidity (recurrence and distant metastasis) of PPB for cases admitted in our institute.

## Results

**Fig. 1:**
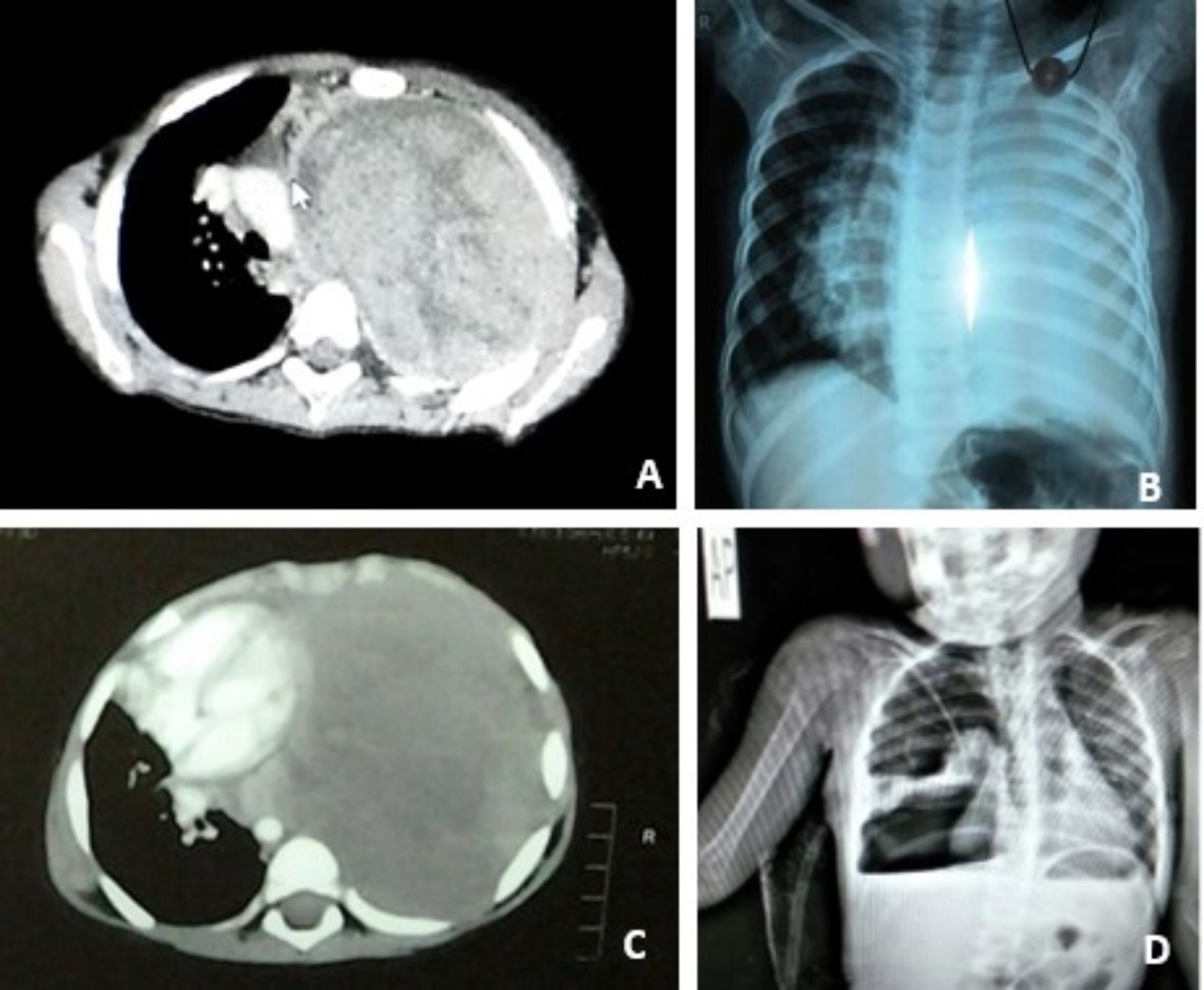
Sample of radiologic findings in the included patients. **(A)** A large sized heterogenous mass on the left side (Case 2). **(B)** Opacification of the left hemithorax (Case 3). **(C)** CT showing homogenous mediastinal mass (Case 3). **(D)** PXR showing hydro-pneumothorax on the right side (Case 4)

During this period, 4 children with PPB were treated in our institution. There were 3 girls and a boy, and the mean age at diagnosis was 3.8 ± 1.1 years (median, 3 years; range, 2.5 to 5 years). No family history of any lung tumors or any relevant conditions. Clinically, patients presented with manifestations ranging from respiratory distress, fever to obstructive shock. Radiologically, 2 cases were presented with mediastinal mass and the other 2 presented with pneumothorax (Fig. 1). None of the cases were diagnosed pre-operatively (Table 1).

**Table 1:** Demographic data, clinical presentation and imaging of the studied patients

Patient	Age (years)	Gender	Clinical presentation	Imaging
1	3	F	sudden onset of dyspnea, cough and chest pain.	CXR: large left pneumothorax with total lung collapse CT: encysted pneumothorax and partial lung collapse
2	5	F	severe respiratory distress, hypotension, tachycardia and elevated jugular venous pressure (obstructive shock)	CXR: total homogenous opacification of the entire left hemithorax with a significant mediastinal shift to the opposite side CT: Huge mediastinal mass
3	3	M	-Low grade fever, tachypnea and tachycardia (suspected empyema) -Drain: only 100 ml serosanginous fluid	CXR: total opacification of the left hemithorax. CT: huge intrathoracic mass, excluding the previous diagnosis
4	2.5	F	-Respiratory distress, tachycardia -Drain: no improvement	CXR: large pneumothorax with total lung collapse CT: hydro-pneumothorax and total lung collapse

**Fig. 2:**
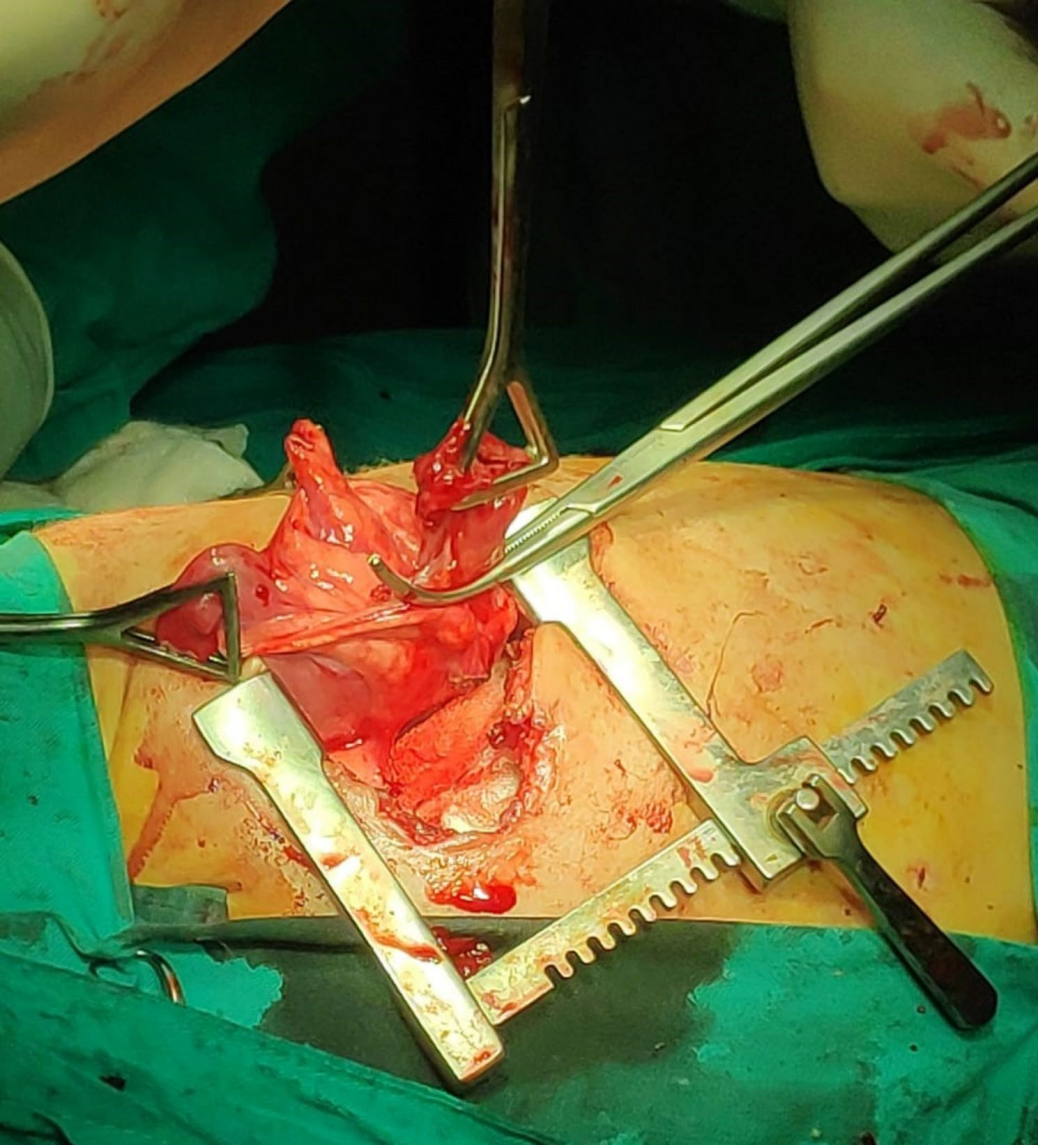
Intraoperative photo of a left sided lesion where a cystic lesion which was responsible for pneumothorax and air leak was clamped before attempting wedge resection. The lesion was proved to be cystic type of PPB (type I)

Intraoperatively, left lung was involved in three-quarters of the cases (2 lower, 1 upper), while only one case had PPB in the right lung (upper lobe). Cystic lung lesion was detected in 2 cases, whilst the other 2 showed large-sized mediastinal mass. One of them was friable, highly vascular and had both cystic and solid components, while the other contained gelatinous material. Wedge resection was done in all cases with the exception of the case that showed huge friable and vascular mass (underwent lobectomy) (Fig. 2) (Table 2).

**Table 2:** Intra-operative details of the studied patients

Patient	Location	Intra-operative finding	Procedure
1	Left (lower)	A 2 cm lung cyst	Wedge resection
2	Left (upper)	Huge soft tissue mass (friable, highly vascular and had both cystic and solid components	Left upper lobectomy
3	Left (lower)	Mass containing gelatinous material	Wedge resection
4	Right (upper)	A small lung cyst	Wedge resection

**Fig. 3:**
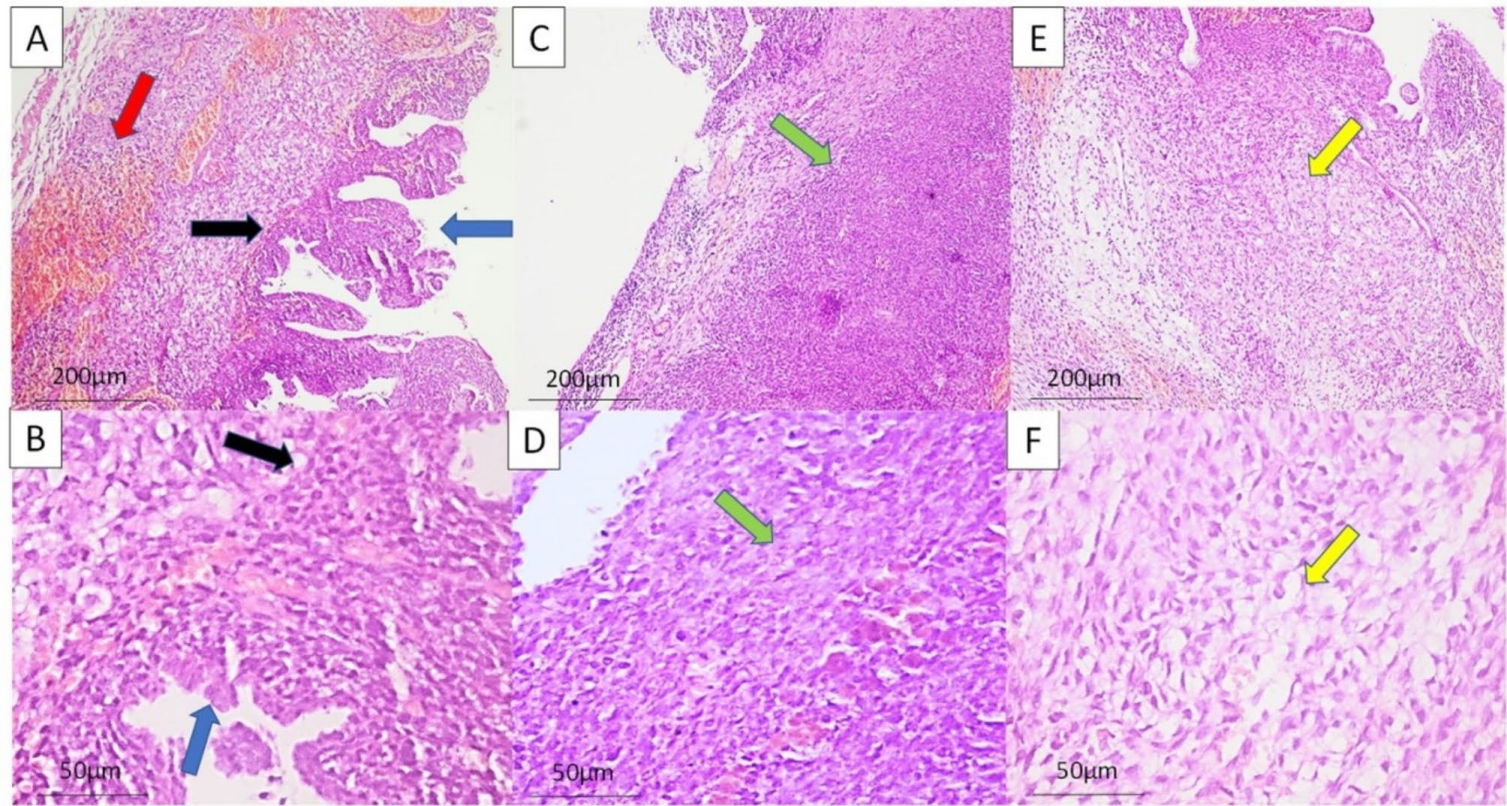
Cystic lesion lined by epithelial cells focally thrown into papillary structures (blue arrow). The cystic septa are thickened by neoplastic masses. The subepithelial areas show condensed primitive blastemal cells (black arrow), with hemorrhagic foci (red arrow) (**A**, H&E, x100). The surface lining is formed of ciliated columnar epithelium (blue arrow), the blastemal cells (black arrow) are small and round with pleomorphic nuclei, little cytoplasm and brisky mitotic figures with scattered large cells (**B**, H&E, x400). Other areas show bundles of spindled sarcomatous cells (green arrow) (**C**, H&E, x100). The spindled cells (green arrow) show pleomorphic features with few large rhabdomyoblast-like cells having eosinophilic cytoplasm (**D**, H&E, x400). Alternating hypocellular areas with myxoid stroma are frequently noted (yellow arrow) (**E**, H&E, x100). The cells are pleomorphic ovoid or spindled with vacuolated cytoplasm (yellow arrow) (**F**, H&E, x400)

**Fig. 4:**
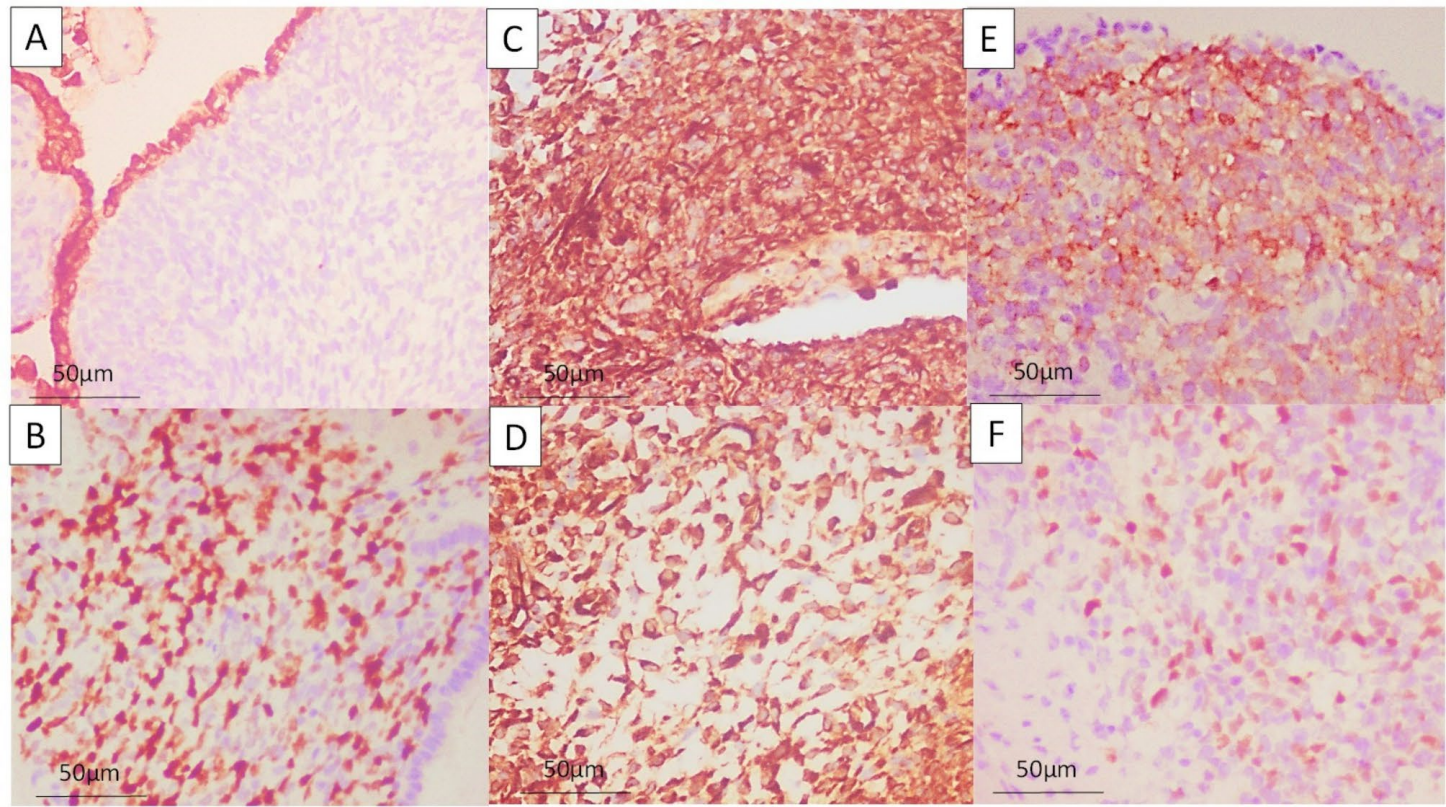
Immunohistochemistry reveals positivity of the cystic epithelial cell lining to pancytokeratin while the neoplastic cells are negative (A, x400). Ki-67 proliferation index average is 75% (B, x400). The neoplastic cells are strongly and diffusely positive to vimentin in hypercellular and hypocellular areas (C, D, x400). The neoplastic cells show positivity to SMA (E, x400) and the scattered rhabdomyoblasts are positive to myogenin (F, x400)

Histologically, all lesions were revealed to be PPB. One lesion proved to be type I, 2 were type II and one was type III (Fig. 3). IHC was done in all cases, showing positivity to vimentin, SMA with one case showing few scattered rhabdomyoblasts that are positive for myogenin. Unfortunately, molecular studies are not present in our country (Fig. 4). All cases underwent chemotherapy using the combination of vincristine, Adriamycin and cyclophosphamide (VAC regimen). Recurrence was detected in a type II case, around 2 years after operation, and the other type II case developed brain metastasis that was discovered 3 years after operation. Type I case showed no local or distant metastasis (Table 3).

**Table 3:** Final diagnosis and post-operative follow-up and outcomes of the studied patients

Patient	Type	Recurrence	Time to recurrence	Outcome	Follow-up years
1	II	(+)	2 years	Receiving chemo	2 Y 3 M
2	III	(-)	--	Free	3 Y
3	II	(-)	--	Brain metastasis	4 Y
4	I	(-)	--	Free	4 Y

## Discussion

The majority of lung cysts are discovered incidentally and previously they were closely followed-up due to the benign nature of most of these lesions [8]. Nevertheless, with the rising number of reports describing PPB, the latter should be considered during the evaluation of patients presenting with cystic lung lesions. It is currently well established that many cystic lung pathologies, e.g. CPAM, have a high chance of developing malignancy and the most common of which is PPB [8–10]. Hence, it was described that it should be included especially in patients with a positive family history of pediatric or unusual malignancy or those showing multiple cystic lesions involving the lung [11].

This report describes a case series of 4 cases of PPB in a tertiary-care hospital. Unfortunately, none of these cases were diagnosed preoperatively because all of them presented with severe respiratory distress resulting from a mass effect or a pneumothorax. This results in lack of full work-up necessary for the accurate diagnosis of these conditions. Consequently, the management was not optimum especially in advanced tumors where more radical excision is needed and an early adjuvant chemotherapy is required.

It is evident that early and appropriate diagnosis definitely improves the prognosis of PPB patients because it hinders its progression into a more aggressive histopathologic type [8, 11, 12]. According to a study by Messinger et al., the progression of a type I PPB to a type II or III preoperatively results to the drop of the 5-year overall survival from 85% in type I into 71% and 53% in type II and III, respectively [1]. Regrettably, recurrent or metastatic PPB can develop even after complete surgical removal of the tumor and the commencement of adjuvant chemotherapy. Hence, it is crucial to monitor those patients closely during the follow-up period [12, 13].

The mainstay treatment for PPB is excision via thoracotomy, although VATS was reported to be used as an initial intervention method in patients presenting with cystic lung disease or pneumothorax [14]. According to the most recent guidelines, treatment differs according to the type of PPB. Type I, for instance, require surgical resection to prevent its progression into the more aggressive type II or III. Adjuvant chemotherapy is rarely required unless subtotal resection was performed [1]. On the other hand, due to the high risk of recurrence and metastasis with type II and III, a more aggressive intervention is needed such as lobectomy or even pneumonectomy if needed to be followed by a high-dose adjuvant chemotherapy [15].

The role of adjuvant radiotherapy in cases of PPB is still equivocal. Theoretically, radiotherapy in the dose of 30–54.8 Gy can be delivered in aiming to targeting the residual tumor in conjunction with chemotherapy [16]. However, because radiation may cause long-term heart damage, it has been recommended that only selected high-risk individuals can receive radiation therapy [17]. Additionally, as per study performed on 50 PPB patients, the 16 patients who received adjuvant radiotherapy did not show any advantage in survival [7].

In our patients, wedge excision was enough for the patient presented with type I. There was no evidence of recurrence or metastasis after 4 years of follow-up. Although type III is the most aggressive form, no recurrence or metastasis was detected in the patient presented with this type. This may be attributed to the more aggressive intervention done intraoperatively (lobectomy) and early start of chemotherapy. However, this patient will still require close follow-up. Local recurrence was found in one of the type II patients, whilst brain metastasis was detected in the other one. This is mostly because of the less radical resection done. Both were managed by wedge excision, though according to the guidelines, we believed a lobectomy seemed to be a better option. Thus, from our prospective, if a solid component was found in admixed within a cystic lesion, a more aggressive therapy should be performed as this raises the suspicion of the more aggressive forms of BBP. Otherwise, recurrence or distant metastasis will occur as encountered in our 2 cases presented by type II.

## Conclusion

To sum up, a prompt preoperative diagnosis and workup of cases of PPB is crucial to enable optimal intervention intraoperatively and early postoperative treatment. Although it is uncommon, PPB should be considered in the differential diagnosis of cystic lung lesions.

## Data Availability

No datasets were generated or analysed during the current study.
